# Impacts of past occupational injury and long-duration compensated work disability on future hospital admissions.

**DOI:** 10.23889/ijpds.v7i3.1973

**Published:** 2022-08-25

**Authors:** Daniel Griffiths, Michael Di Donato, Tyler Lane, Shannon Gray

**Affiliations:** 1 Monash University; 2 University of Toronto

## Objectives

To investigate changes in the prevalence and nature of hospital admissions towards the end of long-duration workers’ compensation claims (>2 years), and afterwards. To examine differences in hospitalisation when workers’ compensation claims end due to either a 260-week duration limit, or otherwise, and comparisons with hospitalisations of a community comparator.

## Approach

A retrospective cohort study examined 2475 workers, termed the s39 group, whose workers’ compensation ceased due to a 260-week limit in 2017/2018 under s39(1) of the Workers’ Compensation Act New South Wales 2012 legislative amendments (Australia). Comparator groups were injured workers with long-duration claims whose compensation ceased independently of s39 (termed the injured control group, N=3626) and a community group (N=8485). Workers’ compensation records were linked to national social security payments, and hospital admissions. Outcomes describe the prevalence and diagnostic categories of hospital admissions 12 months before, and after, the cessation of workers’ compensation stopped payments.

## Results

Musculoskeletal health conditions were common diagnoses in overnight hospitalisations for injured workers. Single-day hospital care for mental health disorders were more common for injured workers (17% of same-day admissions) than for members of a community control (3% of same-day admissions) across two years. Exiting the workers’ compensation scheme is associated with significantly fewer annual hospital admissions for the injured control group (OR 0.76), but not for the s39 group (OR 1.01). Injured workers with long-duration compensated work disability were admitted to hospital more often than the community comparator group during the year after workers’ compensation stops (s39 group: OR 1.55, injured control group: OR 1.30). Across all study groups, hospital admission was more common for people receiving disability social security benefits and older age groups.

## Conclusion

Policy change in the New South Wales workers’ compensation system introduced a 260-week limit on compensation, leaving workers with an elevated need for hospital care after their compensation ended. Welfare policies that disrupt key determinants of health require dedicated inter-agency provisions to support the elevated health needs of those affected.

